# Supplementary figures and images for: Integrated bioinformatics analysis reveals dynamic candidate genes and signaling pathways involved in the progression and prognosis of diffuse large B-cell lymphoma
Source: PeerJ. 2021 Nov 2;9:e12394. doi: 10.7717/peerj.12394 (PMC8570165; doi:10.7717/peerj.12394)

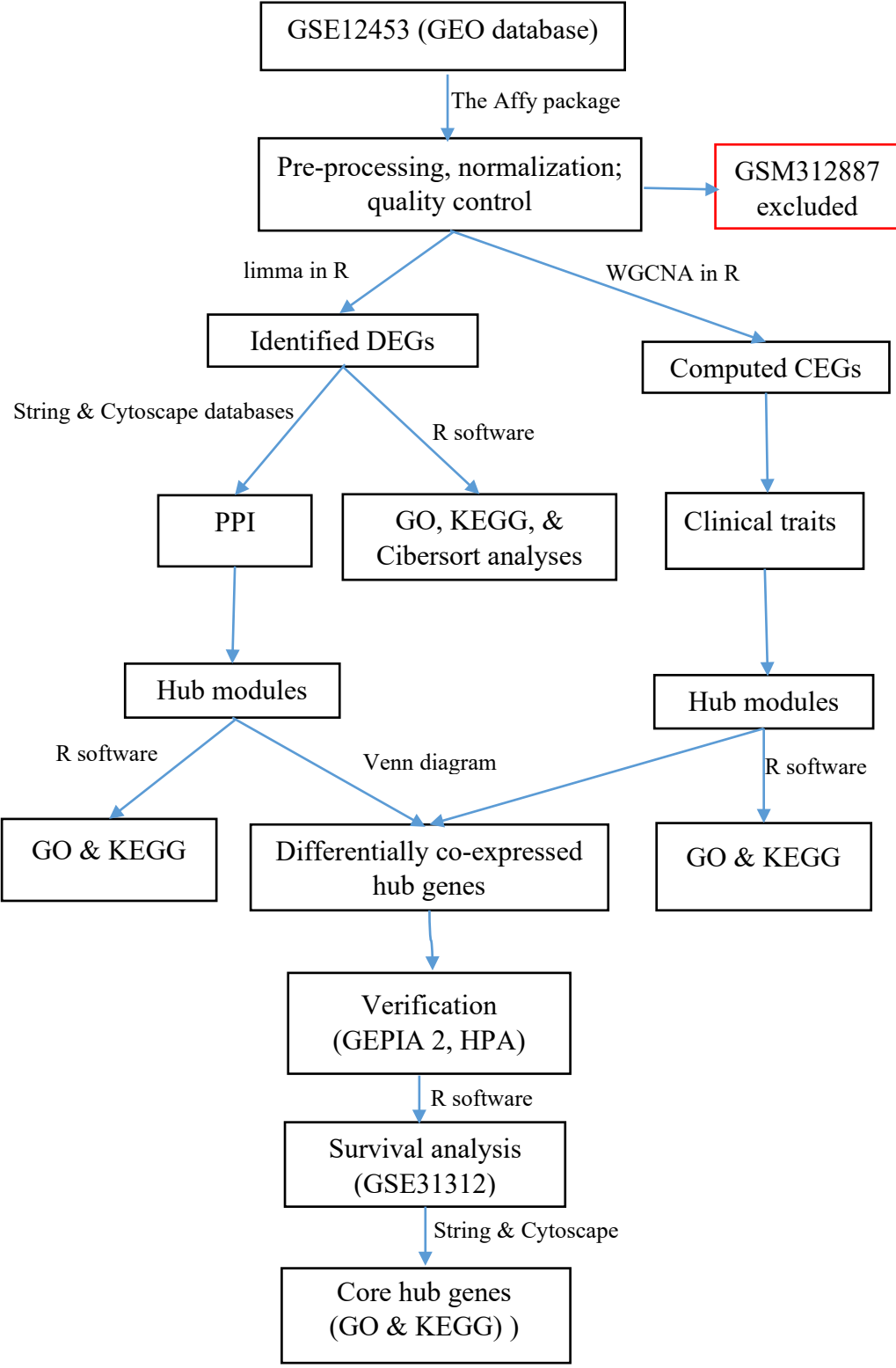

Supplement: Supplemental Information 1 — PPI: protein-protein interaction; DEGs: differentially expressed genes; CEGs: co-expressed genes; HPA: the human protein atlas. [file peerj-09-12394-s001.pdf]

A

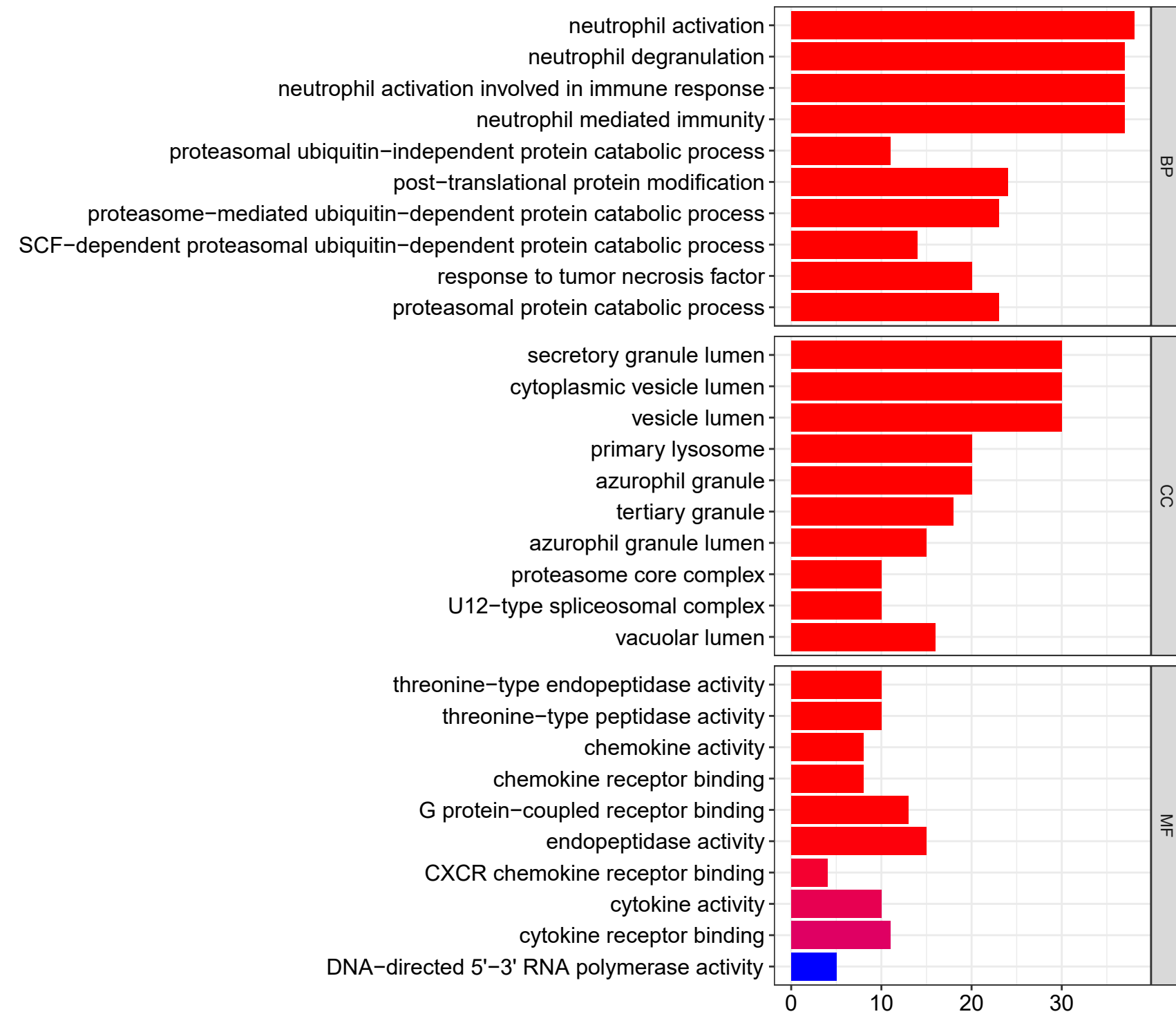

B

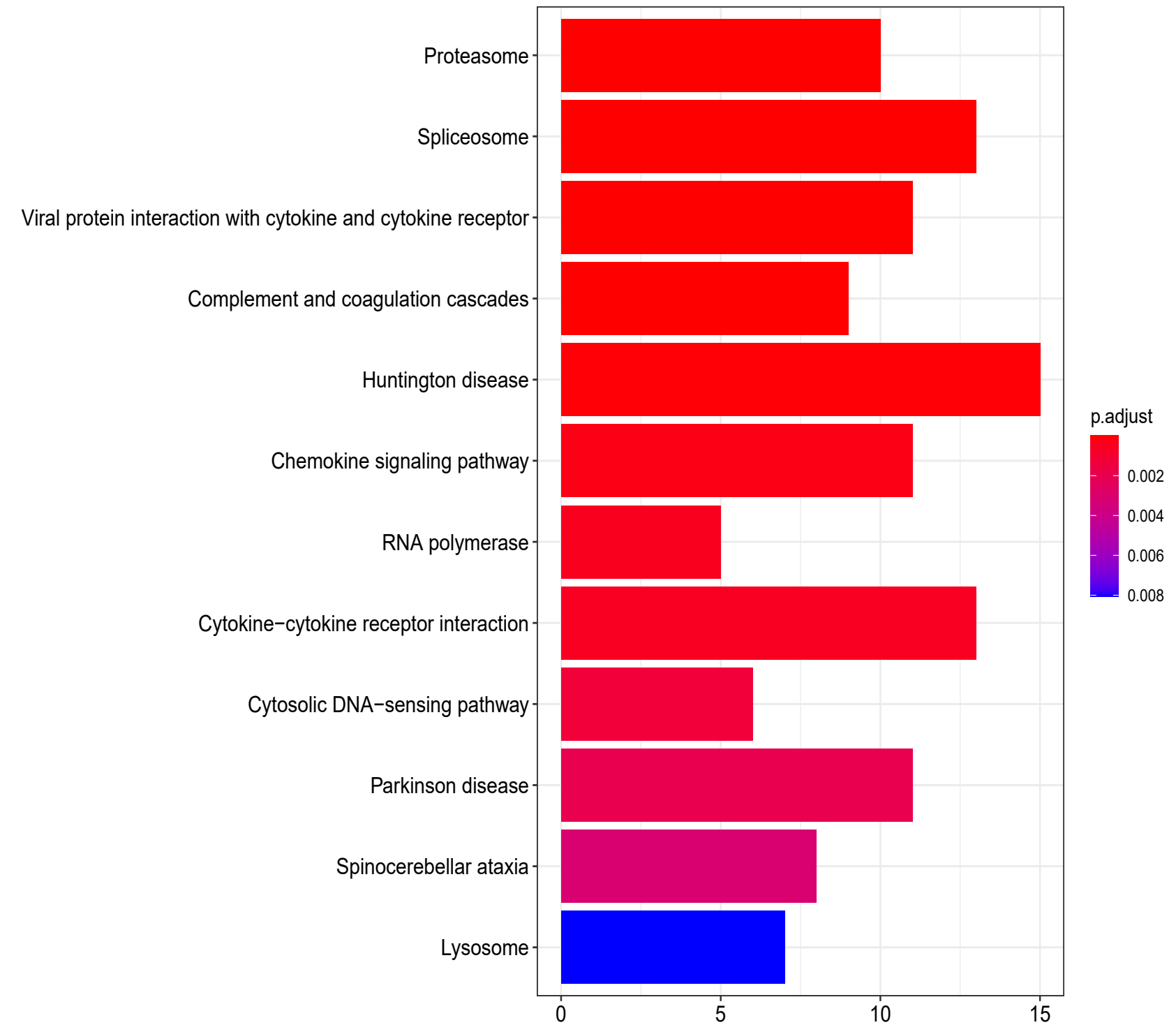

Supplement: Supplemental Information 2 — (A) GO analysis; the top 10 significantly enriched GO terms of intersecting DEGs, p < 0.05. (B) The top 12 significantly enriched KEGG pathways of intersecting DEGs, p < 0.05. The horizontal axis is the number of intersecting DEGs involved in the terms or pathways; the vertical axis shows the terms or pathway names. KEGG (Kyoto encyclopedia of genes and genomes). [file peerj-09-12394-s002.pdf]

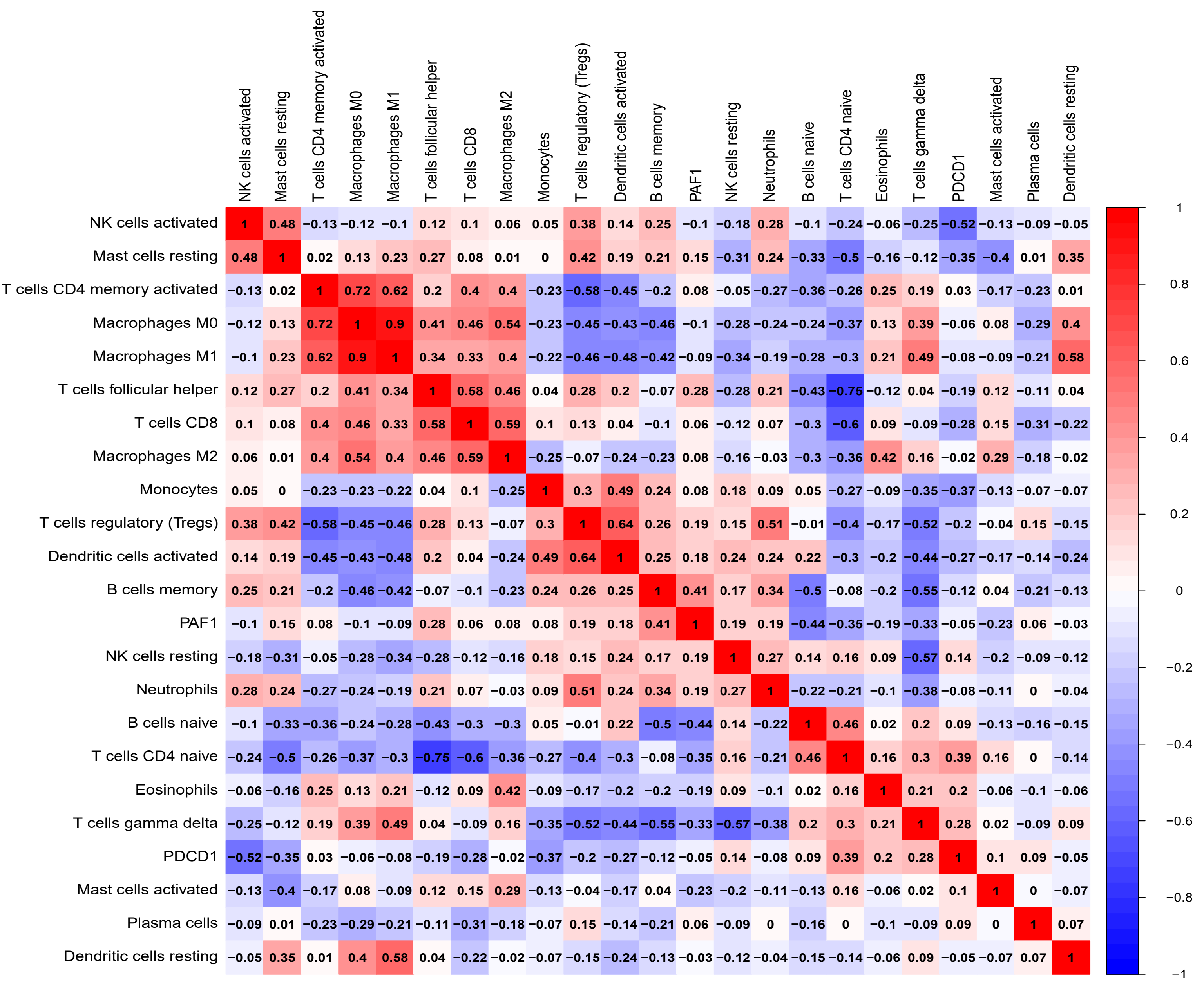

Supplement: Supplemental Information 3 — x-and y-axes both represent tumor-infiltrating immune cells. Color legend: red (positive) and blue (negative) correlations. [file peerj-09-12394-s003.pdf]

A

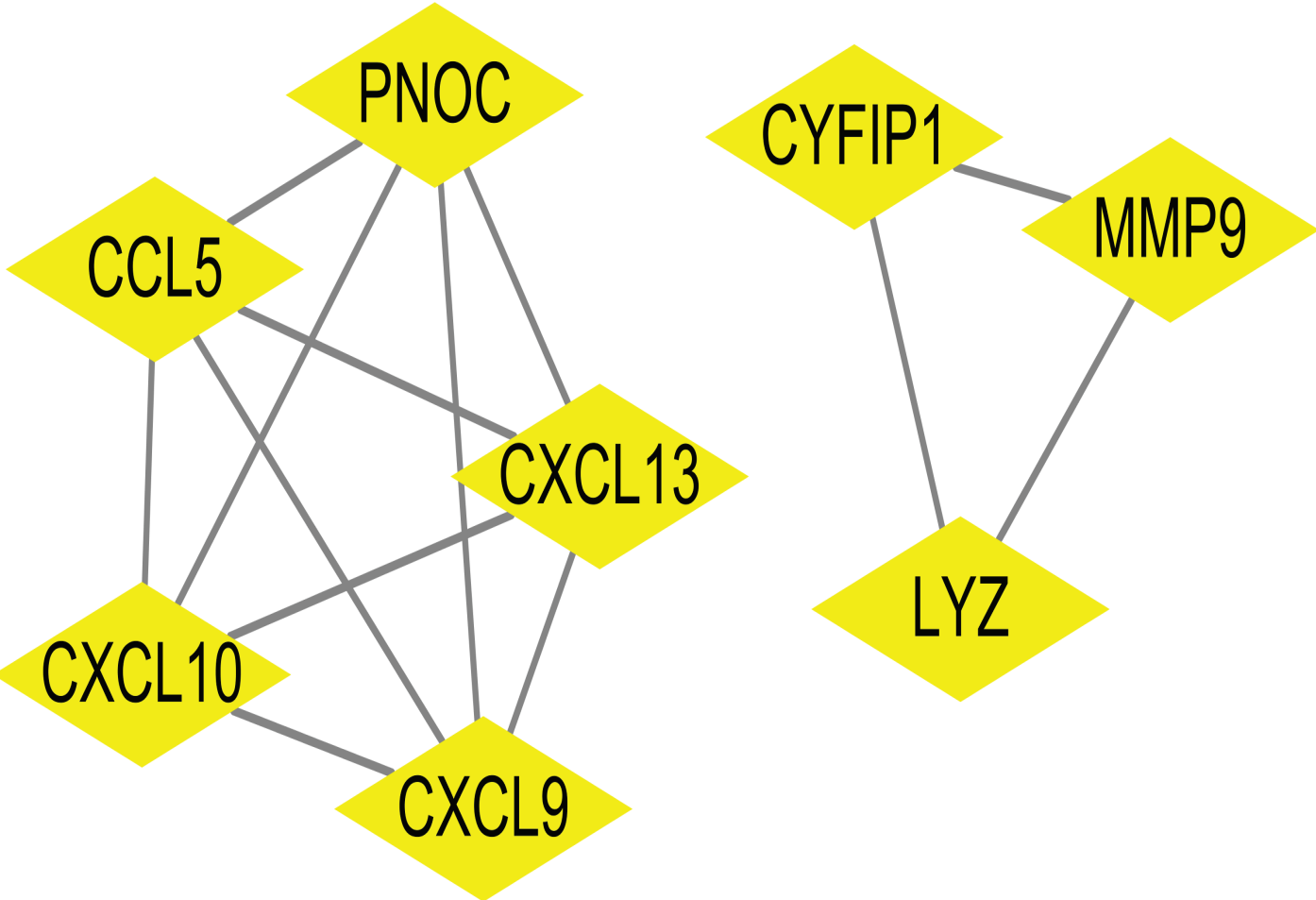

B

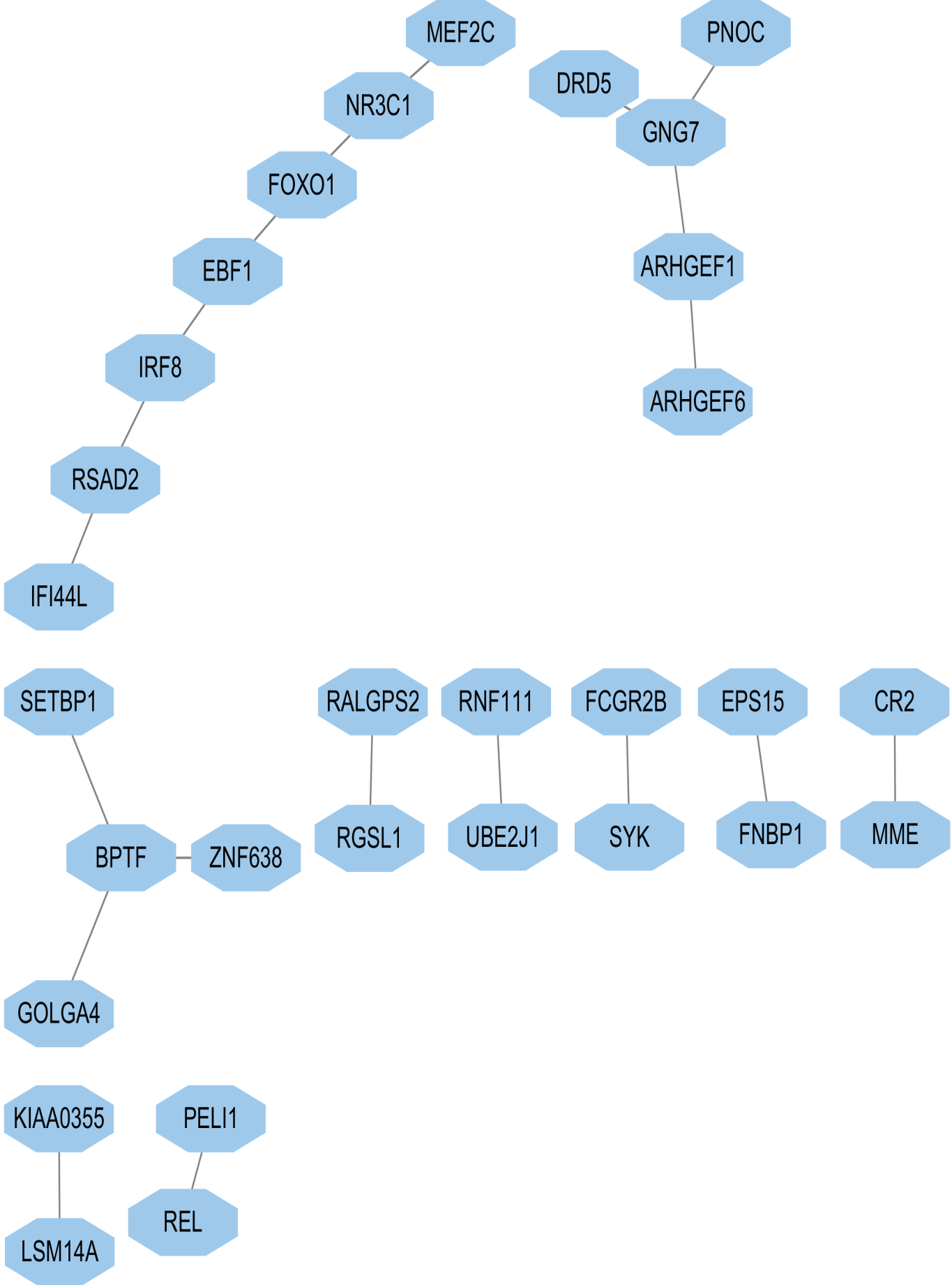

C

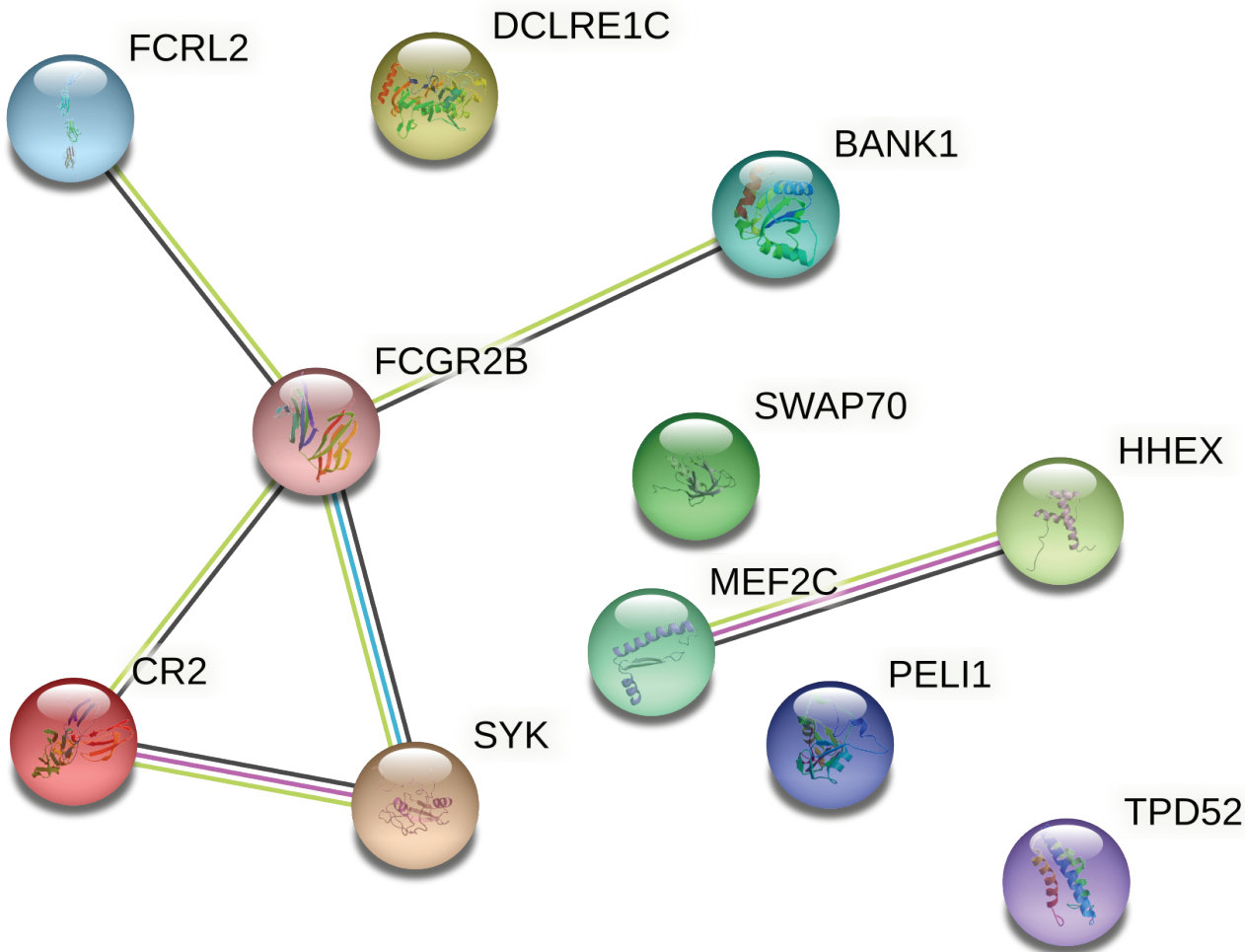

Supplement: Supplemental Information 4 — (A) PPI for the top 30 up-and 30 down-regulated DEGs ranked by highest log2FC at the highest confidence of 0.9. (B) PPI for all downregulated genes at high confidence of 0.7 with disconnected nodes in the network hidden (it yielded 30 nodes, 20 edges). (C) PPI of the downregulated DEGs enriched in B cell activation for GO biological processes (medium confidence, 0.4); false discovery rate, 6.29e−15). [file peerj-09-12394-s004.pdf]

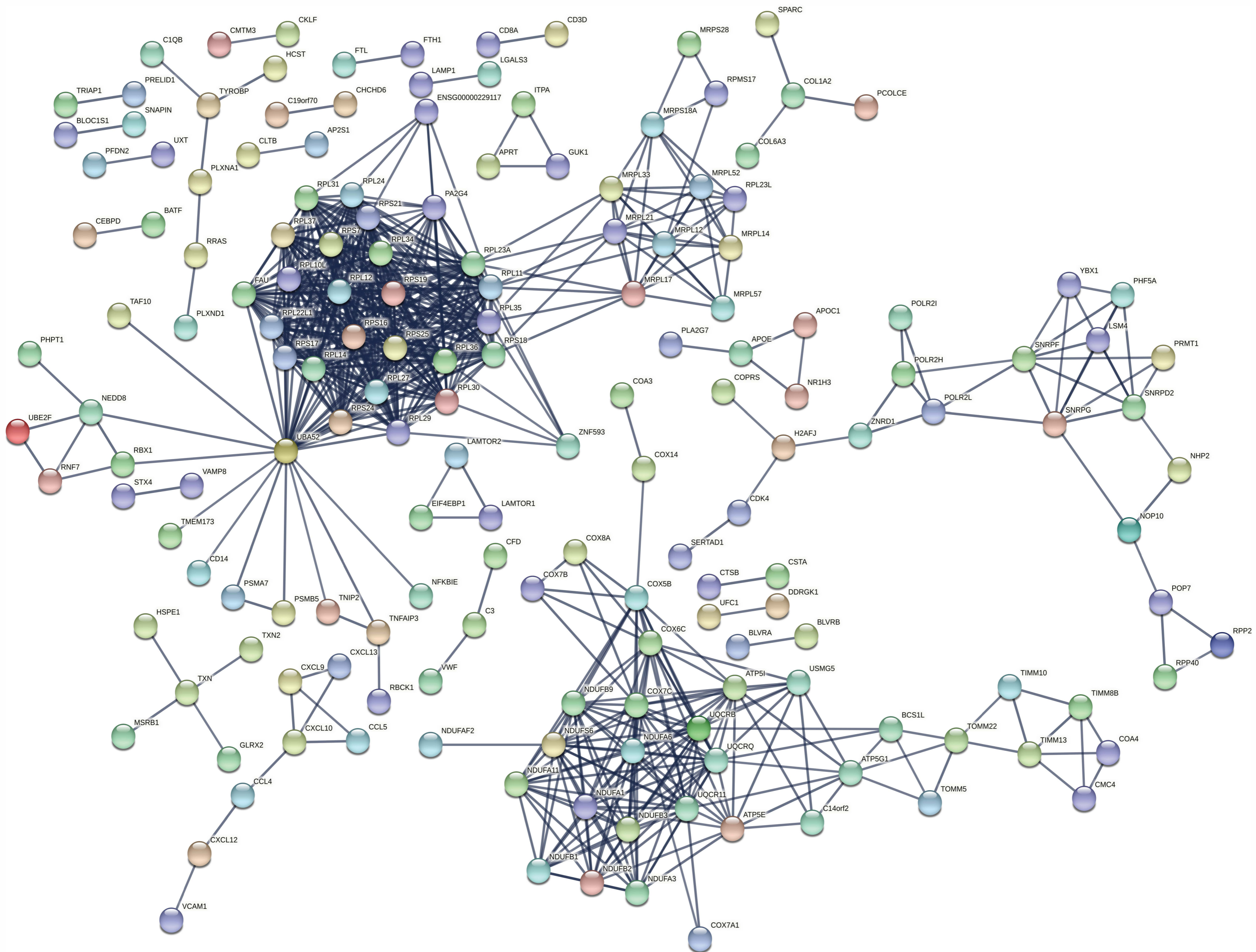

Supplement: Supplemental Information 5 — The PPI network of turquoise, green and dark magenta module genes at high confidence of 0.9 with disconnected nodes hidden. [file peerj-09-12394-s005.pdf]

RPS25

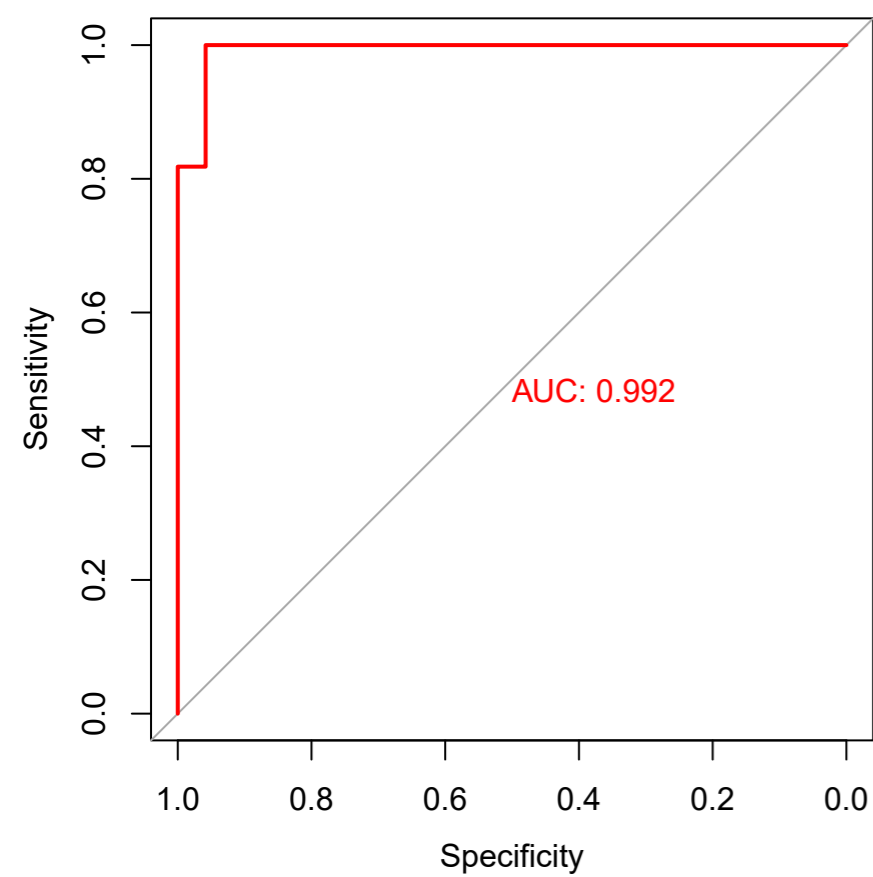

FAU

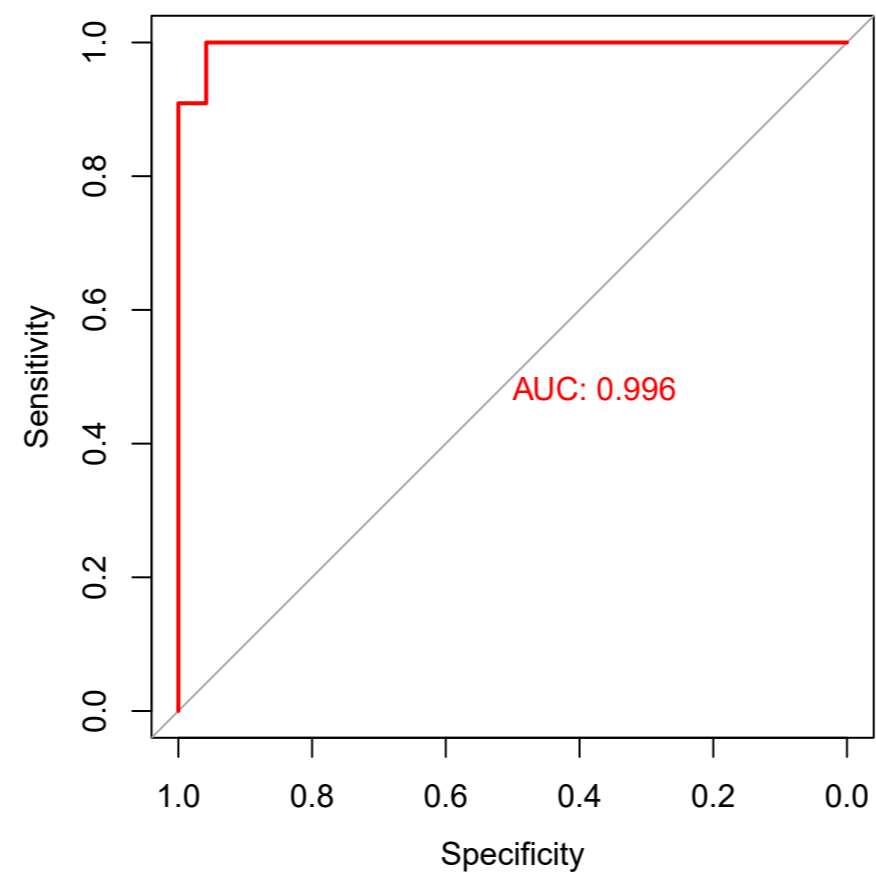

MRPL33

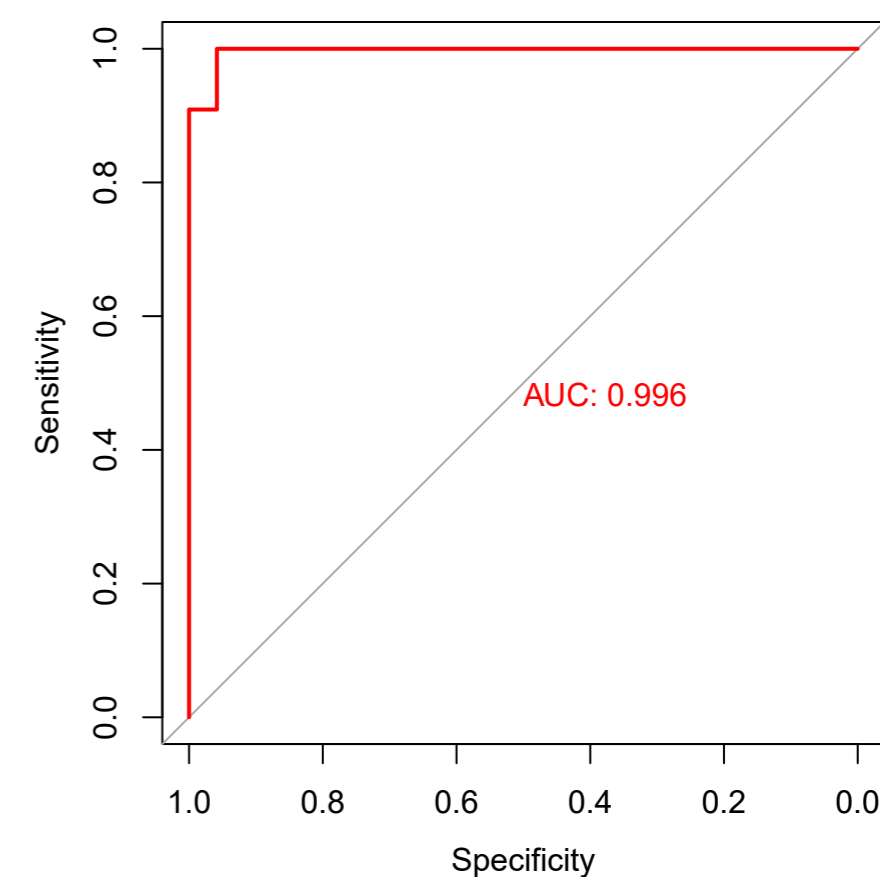

RPL31

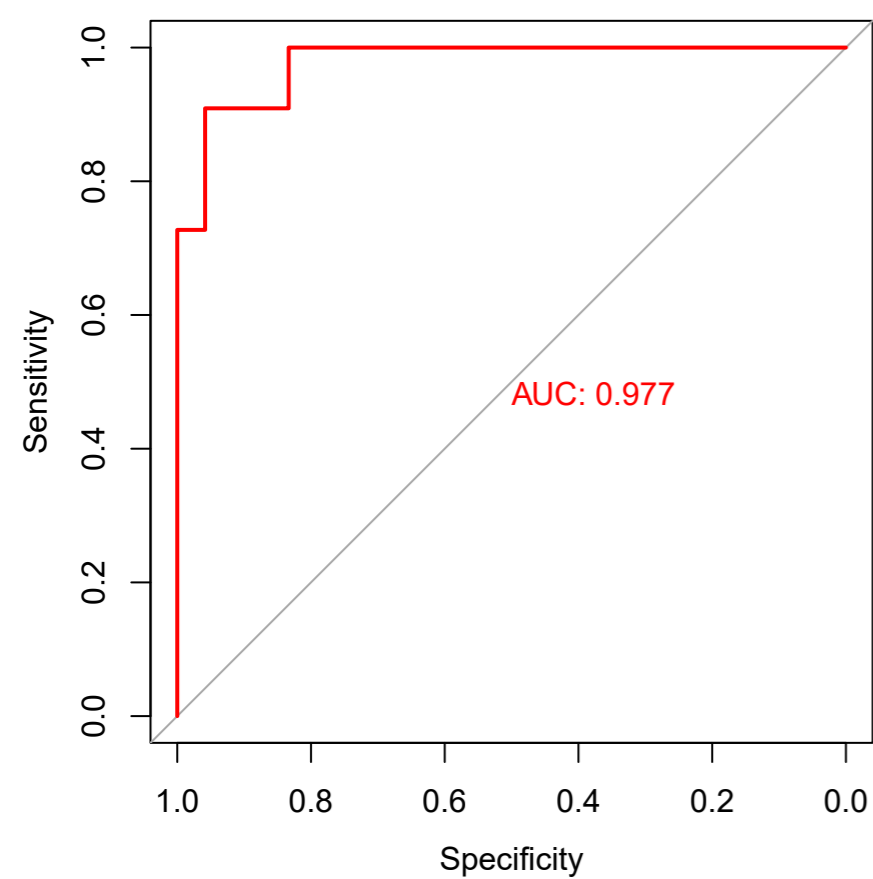

NDUFA6

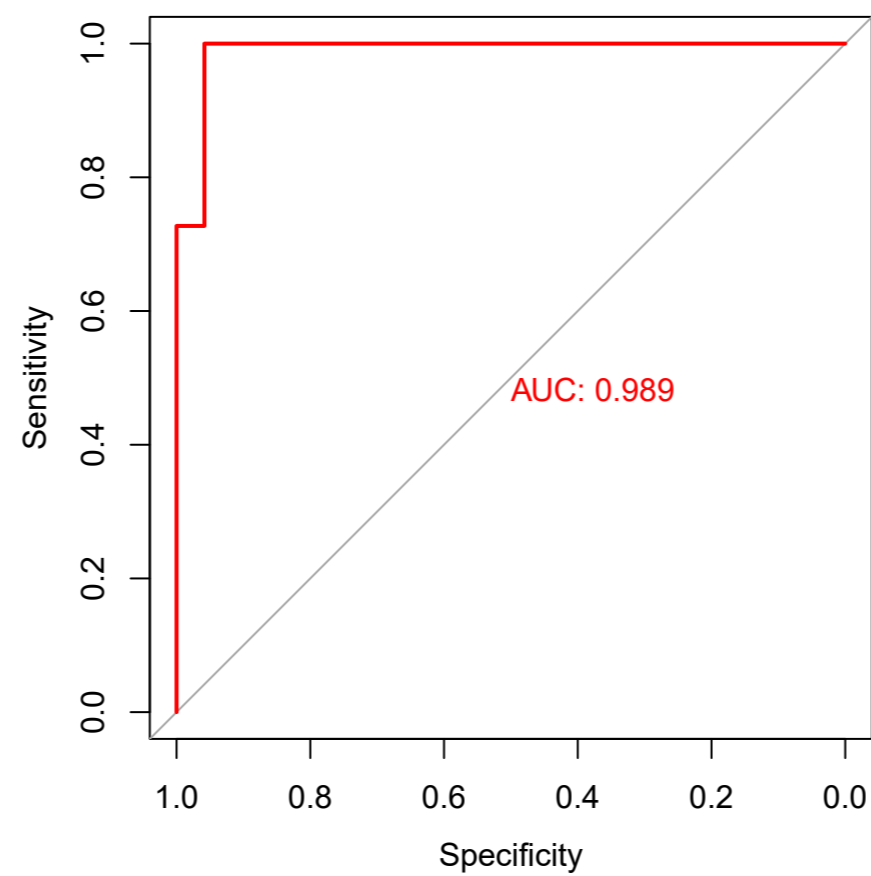

CCL4

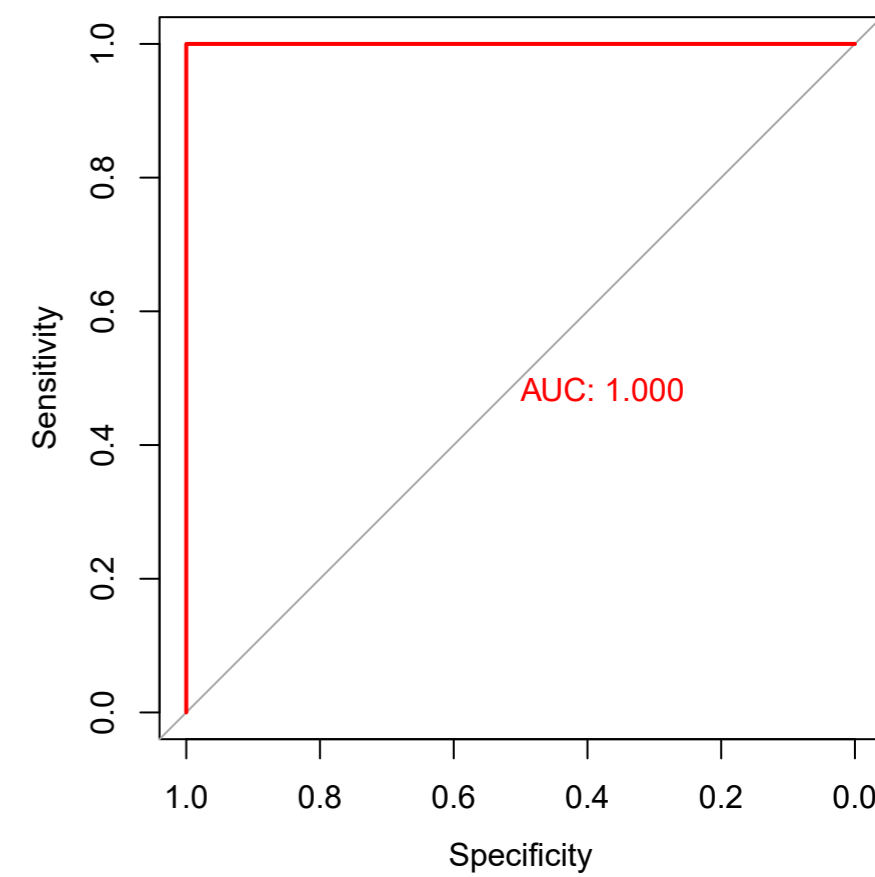

Supplement: Supplemental Information 6 — MRPS28, RPL22L1, RPL30, and RPS17 each had an AUC of 0.989. Also, CXCL9, HEBP1, RPL11, RPS21 and RPS 24 each had an AUC of 1.00. The area under curve (AUC). [file peerj-09-12394-s006.pdf]

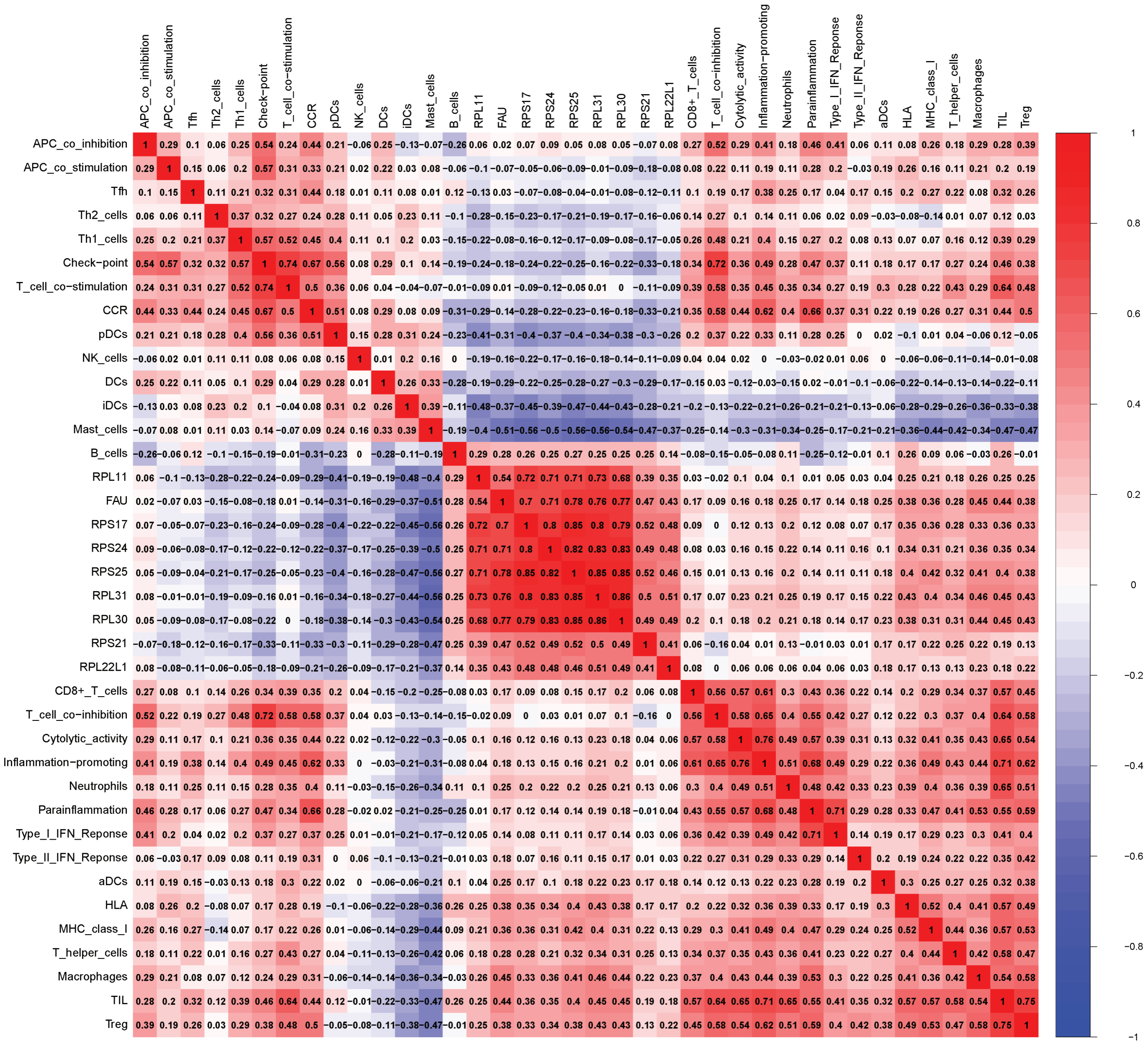

Supplement: Supplemental Information 8 [file peerj-09-12394-s008.pdf]
