# Supplementary material for: Integrated bioinformatics analysis reveals dynamic candidate genes and signaling pathways involved in the progression and prognosis of diffuse large B-cell lymphoma
Source: PeerJ. 2021 Nov 2;9:e12394. doi: 10.7717/peerj.12394 (PMC8570165; doi:10.7717/peerj.12394)
Supplement: Supplemental Information 7 — The averaged protein expression levels of normal tissue (NT)-not otherwise stated (NOS) non-germinal center cells (N-GCC) were relatively lower than the expression levels in some selected tumor tissues. Q: quantity of IHC score; DLBCL: NHL high grade; LG: NHL-low grade; HD-NOS: Hodgkin’s disease-NOS; id: Patient id. Data retrieved from the human protein atlas (https://www.proteinatlas.org/). [file peerj-09-12394-s007.pdf]

HPA003371 (RPS21)

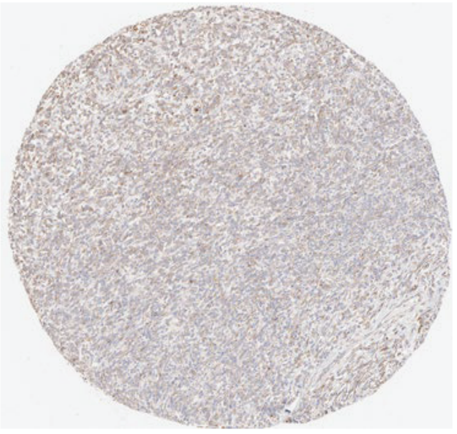

DLBCL; id: 2207; (Q>75%)

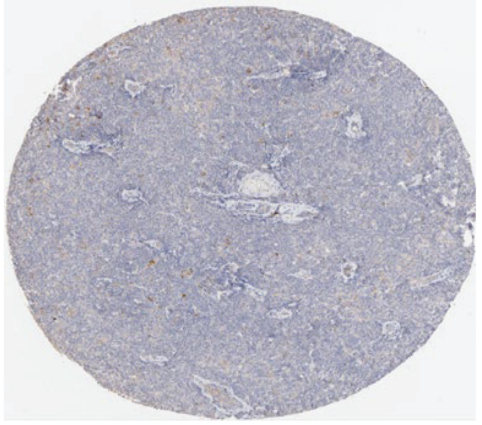

LG; id: 1726; (Q 75%-25%)

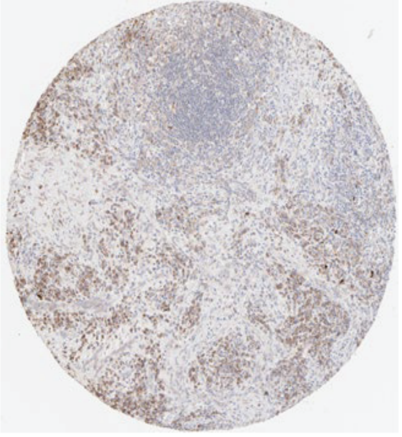

HD-NOS; id: 370; (Q >75%)

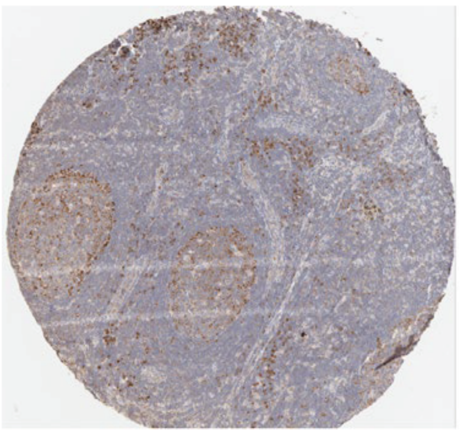

NT-NOS (Q [GCC 75%-25%]; [N-GCC <25%])

HPA027202 (MRPS28)

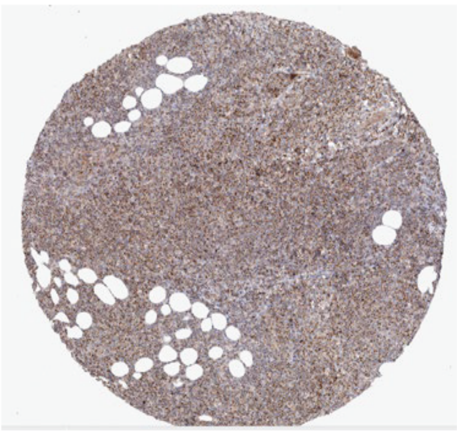

DBCL; id: 1748; (Q>75%)

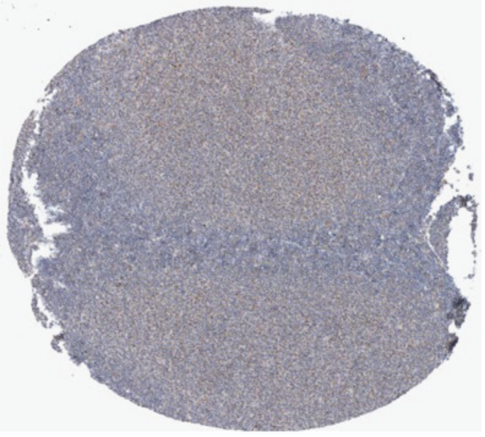

LG; id: 2731; (Q=75%-25%)

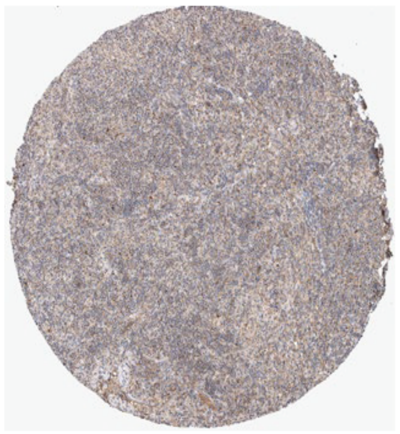

HD-NOS; id: 3232; (Q >75%)

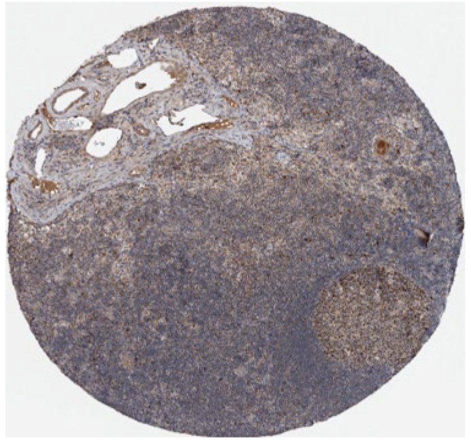

NT-NOS; id: 1690; (Q [GCC >75%]; [N-GCC <25%])

HPA072263 (RPL31)

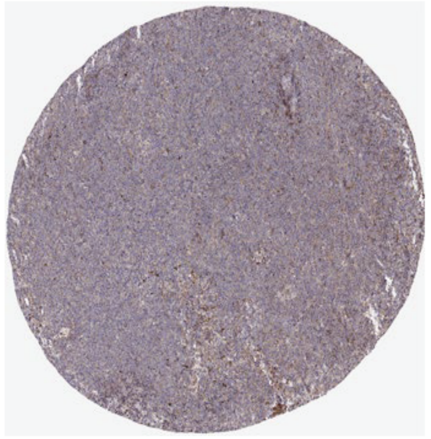

DLBCL; id: 3085; (Q 75%-25%)

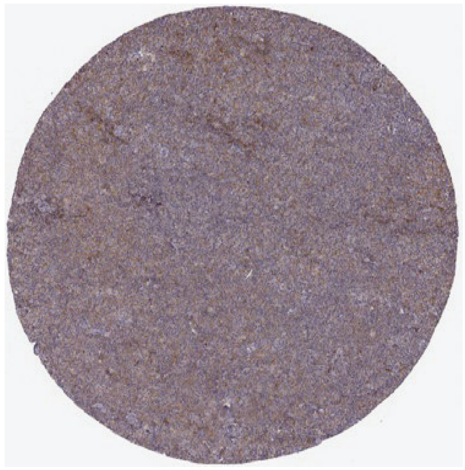

LG; id: 3129; (Q=75%-25%)

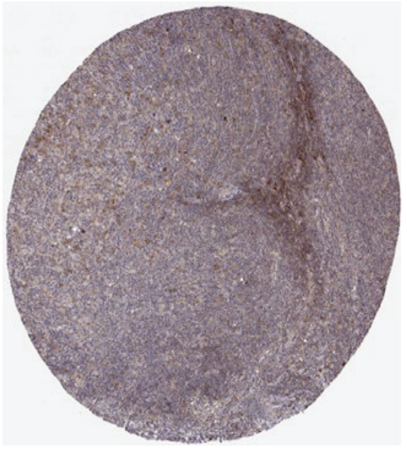

HD-NOS; id: 2532; (Q >75%)

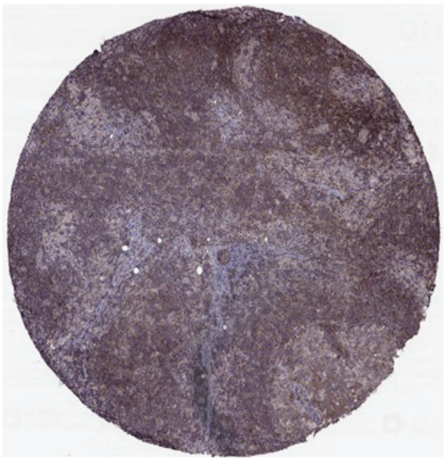

NT-NOS; id: 4449; (Q [GCC >75%]; [N-GCC <25%])

HPA002651 (RPL30)

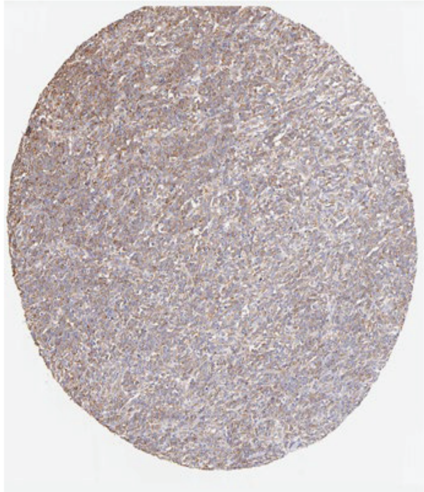

DLBCL; id: 2207; (Q 75%-25%)

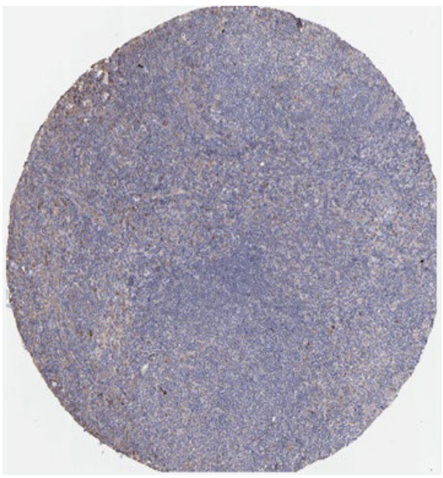

LG; 1618; (Q=75%-25%)

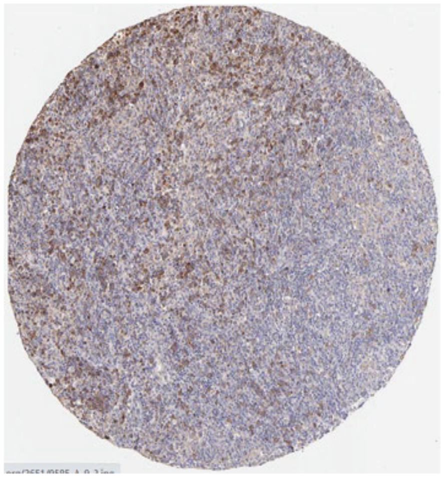

HD-NOS; id: 226; (Q<25%)

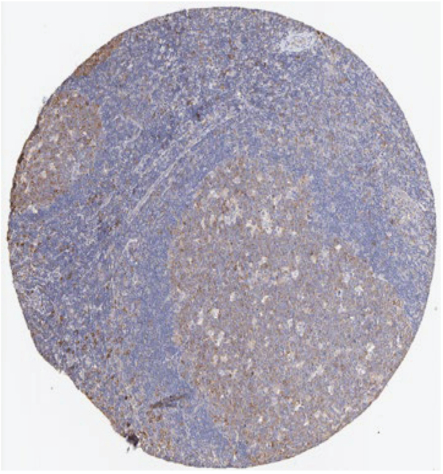

NT-NOS; id: 1636; (Q [GCC >75%]; [N-GCC <25%])
