# Supplementary material for: Integrated bioinformatics analysis reveals dynamic candidate genes and signaling pathways involved in the progression and prognosis of diffuse large B-cell lymphoma
Source: PeerJ. 2021 Nov 2;9:e12394. doi: 10.7717/peerj.12394 (PMC8570165; doi:10.7717/peerj.12394)
Supplement: Supplemental Information 9 — Most of the genes were upregulated in various cancer types. However, some genes were downregulated in some tumors, suggesting that the same gene may have different functions in different cancers. Data were retrieved from the Oncomine database (https://www.oncomine.org/resource/main.html). [file peerj-09-12394-s009.pdf]

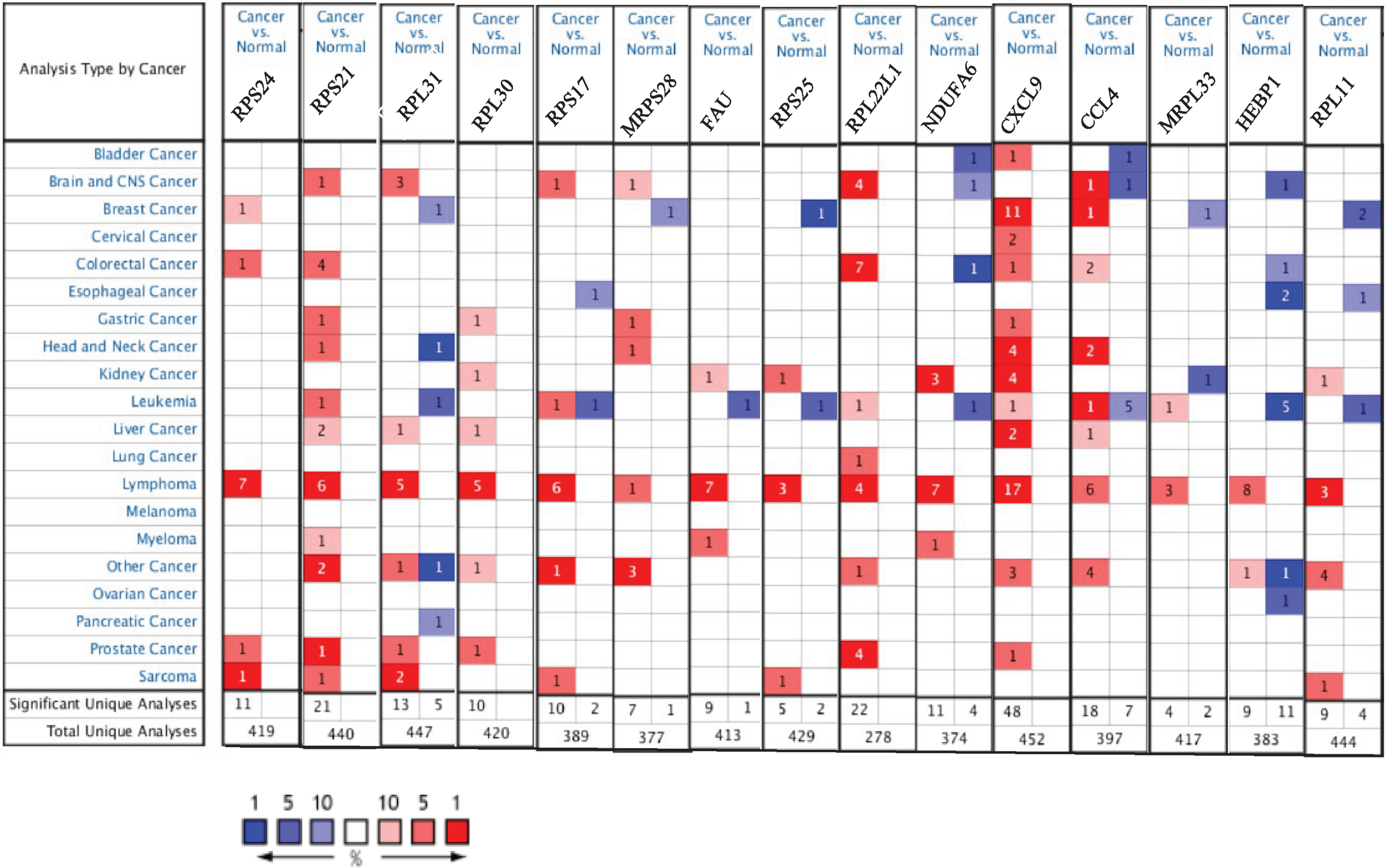

Cell color is determined by the best gene rank percentile for the analyses within the cell.

NOTE: An analysis may be counted in more than one cancer type.
