# Supplementary material for: Integrated bioinformatics analysis reveals dynamic candidate genes and signaling pathways involved in the progression and prognosis of diffuse large B-cell lymphoma
Source: PeerJ. 2021 Nov 2;9:e12394. doi: 10.7717/peerj.12394 (PMC8570165; doi:10.7717/peerj.12394)
Supplement: Supplemental Information 10 — Top 20 hallmark enrichment. ES, Enrichment Score; NES, normalized Enrichment Score; NOM P-val, nominal p value; FDR, false discovery rate >0.25; FWER, familywise-error rate [file peerj-09-12394-s010.docx]

| Enrichment | Size | ES | NES | FDR q-val | FWER p-val |
| --- | --- | --- | --- | --- | --- |
| HALLMARK_COAGULATION | 136 | 0.634 | 1.837 | 0.013 | 0.011 |
| HALLMARK_CHOLESTEROL_HOMEOSTASIS | 71 | 0.621 | 1.811 | 0.010 | 0.015 |
| HALLMARK_XENOBIOTIC_METABOLISM | 193 | 0.496 | 1.777 | 0.015 | 0.028 |
| HALLMARK_ESTROGEN_RESPONSE_LATE | 196 | 0.505 | 1.751 | 0.018 | 0.04 |
| HALLMARK_KRAS_SIGNALING_UP | 192 | 0.505 | 1.726 | 0.022 | 0.054 |
| HALLMARK_EPITHELIAL_MESENCHYMAL_TRANSITION | 195 | 0.583 | 1.717 | 0.022 | 0.062 |
| HALLMARK_APOPTOSIS | 159 | 0.586 | 1.694 | 0.025 | 0.076 |
| HALLMARK_COMPLEMENT | 194 | 0.610 | 1.686 | 0.024 | 0.084 |
| HALLMARK_P53_PATHWAY | 188 | 0.497 | 1.671 | 0.027 | 0.104 |
| HALLMARK_UV_RESPONSE_UP | 153 | 0.504 | 1.658 | 0.029 | 0.122 |
| HALLMARK_IL6_JAK_STAT3_SIGNALING | 87 | 0.613 | 1.607 | 0.052 | 0.192 |
| HALLMARK_FATTY_ACID_METABOLISM | 153 | 0.510 | 1.584 | 0.059 | 0.212 |
| HALLMARK_OXIDATIVE_PHOSPHORYLATION | 180 | 0.679 | 1.584 | 0.055 | 0.212 |
| HALLMARK_MYC_TARGETS_V2 | 56 | 0.642 | 1.577 | 0.055 | 0.224 |
| HALLMARK_ADIPOGENESIS | 189 | 0.517 | 1.570 | 0.055 | 0.235 |
| HALLMARK_INFLAMMATORY_RESPONSE | 198 | 0.559 | 1.570 | 0.051 | 0.237 |
| HALLMARK_ALLOGRAFT_REJECTION | 193 | 0.550 | 1.538 | 0.066 | 0.288 |
| HALLMARK_TNFA_SIGNALING_VIA_NFKB | 194 | 0.562 | 1.537 | 0.064 | 0.291 |
| HALLMARK_NOTCH_SIGNALING | 31 | 0.481 | 1.532 | 0.063 | 0.301 |
| HALLMARK_IL2_STAT5_SIGNALING | 193 | 0.495 | 1.529 | 0.061 | 0.309 |
