# Supplementary material for: Integrated bioinformatics analysis reveals dynamic candidate genes and signaling pathways involved in the progression and prognosis of diffuse large B-cell lymphoma
Source: PeerJ. 2021 Nov 2;9:e12394. doi: 10.7717/peerj.12394 (PMC8570165; doi:10.7717/peerj.12394)
Supplement: Supplemental Information 11 [file peerj-09-12394-s011.docx]

| **Core hub genes** | **OS**  **(p-value < 0.05)** | **PFS**  **(p-value < 0.05)** |
| --- | --- | --- |
| RPS24 | 3.78E-08 | 0.000116 |
| RPS21 | 4.58E-08 | 4.87E-05 |
| RPL31 | 1.20E-05 | 0.008618 |
| RPL30 | 7.21E-05 | 0.002240 |
| RPS17 | 0.000118 | 0.0362949 |
| MRPS28 | 0.000133 | 0.011766 |
| FAU | 0.000561 | 0.019592 |
| RPS25 | 0.000631 | 0.013749 |
| RPL22L1 | 0.000904 | 0.014554 |
| NDUFA6 | 0.007477 | 0.041609 |
| CXCL9 | 0.007678 | 0.013393 |
| CCL4 | 0.012423 | 0.000601 |
| MRPL33 | 0.019831 | 0.000931 |
| HEBP1 | 0.031470 | 0.039510 |
| RPL11 | 0.043610 | 0.035242 |
