# Supplementary material for: Integrated bioinformatics analysis reveals dynamic candidate genes and signaling pathways involved in the progression and prognosis of diffuse large B-cell lymphoma
Source: PeerJ. 2021 Nov 2;9:e12394. doi: 10.7717/peerj.12394 (PMC8570165; doi:10.7717/peerj.12394)
Supplement: Supplemental Information 12 — p-value < 0.01; |Log2FC| > 1. RPL30 and FAU (marked in red) were consistently upregulated in all six tumor types. THYM: Thymoma; TGCT: Testicular Germ Cell Tumors; CHOL: Cholangio carcinoma; LGG: Brain Lower Grade Glioma; GBM: Glioblastoma multiforme; PAAD: Pancreatic adenocarcinoma (http://gepia2.cancer-pku.cn/#analysis) [file peerj-09-12394-s012.docx]

| **S/N** | **Tumor** | **Tumor/normal control samples** | **Upregulated genes** | **Gene list** |
| --- | --- | --- | --- | --- |
| 1 | THYM | 118/339 | 15 | RPS24, RPS21, RPL31, RPL30, RPS17, MRPS28, FAU, RPS25, RPL22L1, NDUFA6, CXCL9, CCL4, MRPL33, HEBP1, RPL11 |
| 2 | TGCT | 137/165 | 11 | RPS24, RPS21, RPL31, RPL30, RPS17, MRPS28, FAU, RPS25, CXCL9, CCL4, RPL11 |
| 3 | CHOL | 36/9 | 10 | RPS24, RPS21, RPL31, RPL30, RPS17, FAU, RPS25, RPL22L1, MRPL33, RPL11 |
| 4 | LGG | 518/207 | 9 | RPS24, RPL30, RPS17, MRPS28, FAU, RPS25, RPL22L1, CCL4, RPL11 |
| 5 | GBM | 163/207 | 9 | RPS21, RPL30, RPS17, MRPS28, FAU, RPL22L1, CXCL9, CCL4, RPL11 |
| 6 | PAAD | 179/171 | 8 | RPS21, RPL30, MRPS28, FAU, NDUFA6, CXCL9, CCL4, MRPL33 |
